# Does the length of dental procedure influence children’s behavior during and after treatment? A systematic review and critical appraisal

**DOI:** 10.15171/joddd.2018.011

**Published:** 2018-03-14

**Authors:** Zahra Jamali, Ebrahim Najafpour, Ziya Ebrahim Adhami, Alireza Sighari Deljavan, Naser Asl Aminabadi, Sajjad Shirazi

**Affiliations:** ^1^Assistant Professor, Department of Oral Medicine, Faculty of Dentistry, Tabriz University of Medical Sciences, Tabriz, Iran; ^2^Associate Professor, Department of Pediatric Dentistry, Faculty of Dentistry, Tabriz University of Medical Sciences, Tabriz, Iran; ^3^Professor, Department of Pediatric Dentistry, Faculty of Dentistry, Tabriz University of Medical Sciences, Tabriz, Iran; ^4^Associate Professor, Department of Pediatric Dentistry, Faculty of Dentistry, Tabriz University of Medical Sciences, Tabriz, Iran; ^5^Postgraduate Student, Department of Oral and Maxillofacial Surgery, Faculty of Dentistry, Islamic Azad University, Isfahan (Khorasgan) Branch, Isfahan, Iran; ^6^Researcher and Lecturer, Dental and Periodontal Research Center, Faculty of Dentistry, Tabriz University of Medical Sciences, Tabriz, Iran; ^7^Research Center of Psychiatry and Behavioral Sciences, Tabriz University of Medical Sciences, Tabriz, Iran

**Keywords:** Dental anxiety, behavior management problems, treatment duration, coping, distress

## Abstract

The aim of this systematic review was to investigate the effect of treatment duration on children’s behavior and/or anxiety in the dental setting. To this end, a systematic search was conducted in Pubmed/Medline and Scopus from 1970 to march 2017 for English language articles that assessed the relationship between dental treatment duration or length, and fear/anxiety or behavior in children aged <12 with no confounding medical and/or psychological history and neuro-psychiatric disabilities. Four studies investigating the effect of treatment duration on children’s behavior during and/or after treatment were included. None of the reviewed studies investigated the effect of treatment duration on children’s dental anxiety or fear. There was a general tendency towards deterioration of children’s behavior with an increase in treatment duration. In conclusion, our results undermine the validity of current suggestions about the appropriate treatment duration. Further clinical trials are needed to establish appropriate treatment duration for more effective behavioral management of pediatric patients during dental proce-dures.

## Introduction

Dental practitioners should consider some key requirements in treatment protocols, including interaction between practitioner, patient and parents; socio-demographic variables and the complexity and duration of treatment.^1^ Children generally differ in their ability to meet practitioner’s demands, and cope with the length of time during which they must be treated. This means that strengthening children’s confidence must be addressed to create optimal treatment by intervening at the level of their coping skills relating to the nature of some specific treatment demands.^2-4^

Although some of the psychophysiological variables which put young children at risk for behavior management problem (BMP) are static and may not be amenable to intervention, appropriate scheduling of appointments and adjusting the treatment plan considering the effect of age and appointment length should be regarded as a part of behavior management strategy in children. In child patients it is important to maintain a balance between the duration of the procedure and efficient behavior management. Shorter appointments have been suggested as a cooperation-enhancing approach for pediatric dental patients.^4,5^ Furthermore, children usually interpret longer treatment sessions as a sign of major problems that might cause significant anxiety leading to the development of behavioral management problems.^4,6^ On the other hand, it is practically impossible to effectively complete many procedures in a short appointment and decreasing the treatment period may be undesirable.^4,6^

Therefore, this work comprehensively reviews the effect of treatment duration on children’s behavior and/or anxiety in the dental setting. The results of this review may help researchers and clinicians distinguish and schedule more appropriate lengths for dental treatments in children.

## Methods

### Search strategy

A systematic search was conducted by searching electronic databases Pubmed/ Medline and Scopus for English language peer-reviewed articles published between 1970 and March 2017 using the search terms (("treatment duration" OR "treatment length" OR "appointment length" OR "dental treatment duration" OR "dental treatment length" OR "dental appointment length" OR "dental anxiety" OR "dental phobia" OR "dental fear" OR "odontophobia" OR "dental distress" OR "dental stress" OR "dentist phobia") AND ("infant" OR "child" OR "adolescent" OR "children" OR "young" OR "young person" OR "minor" OR "pediatric" OR "pediatric")). A database was created for the found records, where they were entered and duplicate entries were removed.

After searching the databases, some recognized pediatric journals in this field, including the International Journal of Pediatric Dentistry, Pediatric Dentistry, The Journal of Clinical Pediatric Dentistry, European Journal of Pediatric Dentistry and Journal of Dentistry for Children, were also hand searched. In addition, the reference lists of selected articles were manually searched in order to complement the search database.

### Inclusion criteria

All the studies that had available abstract in English and assessed dental treatment duration or length, and fear/anxiety or behavior in children aged <12 years old; the participants had no confounding medical and/or psychological history and neuro-psychiatric disabilities that could influence their behavior.

### Exclusion criteria

Papers having any of the below criteria were excluded: Mixed populations (unless specific data were available for the target age group); letters to editor, presentations in conferences, case reports and unpublished papers.

For qualification, all the criteria had to be either clearly mentioned in the study or later represented by the corresponding author; otherwise, the study was excluded from the systematic review. All the papers that passed the abstract screening were retrieved in their complete forms, and data extraction was conducted.

### Data extraction

A standardized data extraction form was developed and pilot-tested. Two reviewers from the team independently applied the inclusion criteria when reviewing the abstracts and complete papers. The initial selection was based on the titles and abstracts of the studies. A third reviewer conducted a random check of approximately 10% of titles and abstracts to check reliability of initial screening. Disagreements were resolved through discussion. If disagreement persisted, the judgment of the third reviewer was decisive.

The following data were then extracted from the articles using the data extraction form: Assessing for dental treatment length; children's behavior, anxiety or fear during/after dental treatment; country and setting where the study was conducted (private office, dental school, clinic etc.); study’s sample size, design and randomization; context including the type of dental procedure and interventions; characteristics of participants, including age and gender; time of assessment of behavior/anxiety/fear/phobia (before, during and/or after treatment).

## Results

**Figure 1:**
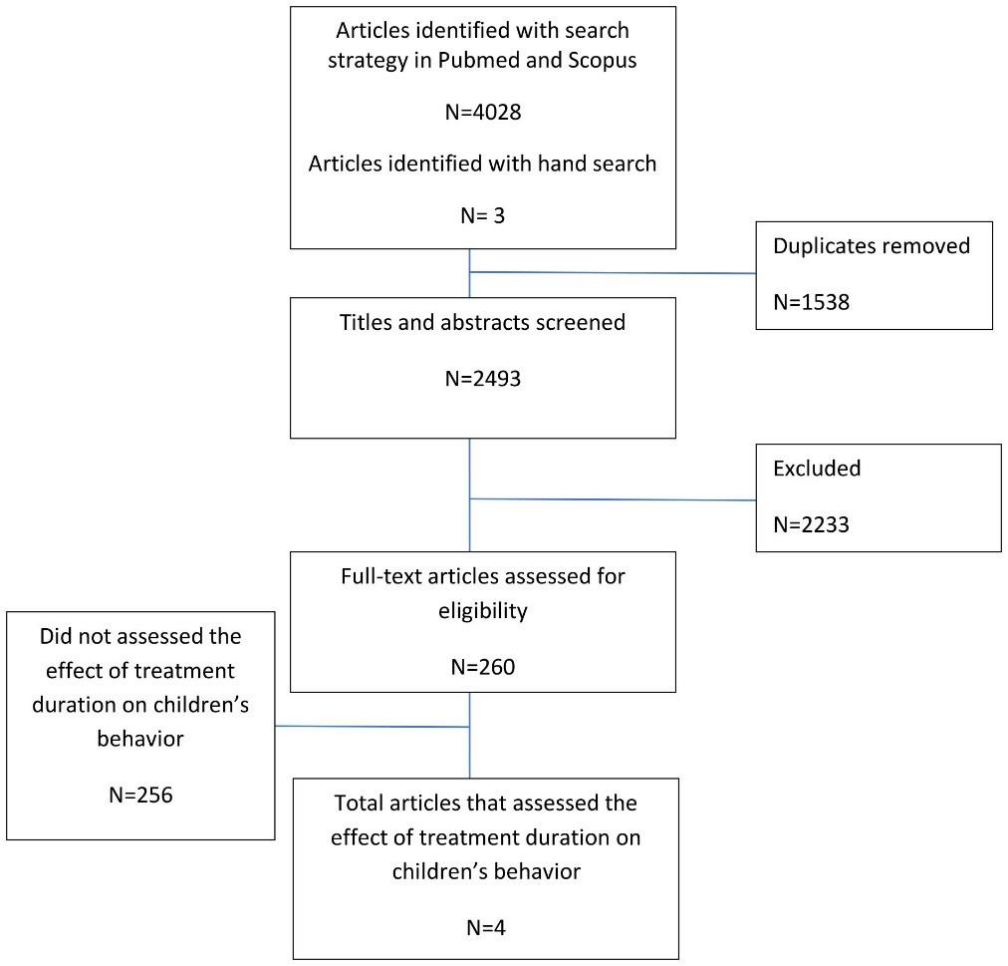
Literature review flow diagram

The search strategy initially returned 2493 identical articles. Three articles were identified by hand search. A review of the titles and abstracts of initial articles yielded 260 studies for further consideration. These articles were reviewed independently by two of the authors to ensure that they met all of the review criteria. Following the reviewers' assessments, four studies met all of the study’s criteria (Figure 1). There was 90% agreement for inclusion when complete papers were reviewed.

All of the included studies were observational, recording behavior of the children during or after treatment. Years of publication ranged from 1966 to 2013, with two of the studies conducted prior to 2000.^5,7^ Two studies were performed in USA,^5,7^ one study in Iran^4^ and one in Israel.^8^ A total of 616 children were observed in these studies with a range of 36 to 450 participants per study. The most common dental procedure reported was cavity preparation and restoration. Preventive resin restoration, prophylaxis and fluoride therapy, pulpotomy and extraction were also reported.^4,5,7,8^ One study provided dental treatment under conscious sedation with oral premedication.^8^ The data extraction for the included studies is presented in Table 1.

**Table 1:** Data extraction table for included studies.

**Reference**	**Context**	**N**	**Age**	**Child measure**		**duration**		**Age differences**	**Relation between Complexity of treatment and behavior**	**Previous calibration of raters/ assessment of inter rater reliability**	**Key findings**
				**during treatment**	**after treatment**	**short**	**long**				
Davidovich 2013^8^	Restorative treatment under conscious sedation with oral premedication.	90	2‒5.5	Behavior **during** treatment was rated as negative (1-3) or positive (4-6), according to Houpt’s scale.	Behavior was ranked as negative when one or more of the following manifestations were observed: crying; aggression; disruptive behavior; temper tantrum; agitation; jerking of the arms and legs; or otherwise, as positive behavior.	15‒30 min	30‒60 min	No	No	Yes	Behavior **during** treatment was associated with treatment duration for both the younger (P<0.001) and older (P<0.04) children. In the younger group, behavior immediately **after treatment** was significantly correlated to treatment duration (P<0.001). In the older group, no significant correlation was found between treatment duration and behavior after treatment (P=0.55).
Getz 1981^7^	restorative treatment for all participants	36	3‒5	Self-developed scale according to Glennon and Weisz’ Preschool Observation Scale of Anxiety		<5 min	5‒10 min	NA	NA	Yes	Length of each phase was found to be significantly related to fear/distress-related behaviors. No relationship was found between child behavior and length of total appointment
Aminabadi 2009^4^	preventive resin restoration, prophylaxis, and fluoride therapy for all participants	450	3‒9	behavior at the end of each treatment period using the sound, eye, and motor (SEM) scale				Yes	NA	Yes	A significant main effect of treatment duration (P<0.001) and age (P<0.001) on behavior.
Lenchner 1966^5^	Restoration in short appointments Pulpotomy and restoration, space maintainer and extraction in long appointments Both type of treatment were provided for all participants	40	3‒6 and 6‒12	dentist’s rating of children’s behavior	Parent’s rating of children’s behavior	<30 min	>45 min	No	NA	-	No difference in children’s behavior between long and short appointments

### Effect of treatment length on children’s behavior dur-ing treatment

Four studies reported the effect of treatment length on children’s behavior during dental treatment. Davidovich et al^8^ reported that behavior during treatment was associated with treatment duration in children aged 2‒5.5 years. Aminabadi et al^4^ reported that treatment duration had a significant and main effect (P<0.001) on children’s behavior. Getz and Weinstein^7^ divided the restorative appointment into six distinct phases and reported that the length of each phase was significantly related to fear/distress-related behaviors (P=0.001). However, no relationship was found between children’s behavior and the total length of the appointment. Lenchner^5^ reported no differences in children’s behavior between long and short appointments or treatments (Table 1).

### Effect of treatment length on children’s behavior after treatment

The effect of treatment length on children’s behavior after dental treatment was investigated in two studies. Lenchner^5^ reported no difference in children’s behavior between long and short appointments. Davidovich et al^9^ reported that behavior immediately after treatment was significantly related to treatment duration in 2‒3.5-year-old children (P<0.001). However, no significant correlation was found between treatment duration and behavior after treatment in 3.5‒5.5-year-old children (Table 1).

### Methods of assessment of children’s behavior

Behavior during treatment was assessed using the sound, eye and motor (SEM) scale,^4^ Houpt’s scale^8^ for general behavior and two self-developed questionnaires.^5,7^ In these studies the dentists rated the children’s behavior during treatment. Children’s behavior after treatment was rated by parents and dentists (Table 1).

#### Effect of treatment length on behavior of children with different ages and genders

Three studies investigated the effect of treatment length on behavior of children with different ages. Aminabadi et al^4^ observed an inverse and significant correlation between children’s age and behavior during treatment in 3‒9-year-old children (P<0.001). Davidovich et al^8^ noted that behavior during treatment was associated with treatment duration in 2‒3.5-year-old (P<0.001) and 3.5‒5.5-year-old children (P<0.04). However, behavior immediately after treatment was significantly correlated with treatment duration only in 2‒3.5-year-old children (P<0.001). In Lenchner’s^5^ study, no difference was observed between the behavior of 3‒6- and 6‒12-year-old children in long and short appointments either during or after treatment.

None of the included studies differentially investigated the effect of treatment length on behavior of boys and girls.

#### Relation between complexity of treatment and children’s behavior

Davidovich et al^8^ considered the effect of treatments with differing complexity on children’s behavior during and after treatment. No significant relationship was found between different treatments and children’s behavior.

#### Data analysis approaches

All the studies included in this review adopted traditional analysis methods. Aminabadi et al^4^ used repeated measures ANOVA and post hoc analyses. Davidovich et al^8^ incorporated chi-squared or Fisher’s exact test, 2-sample t-test and logistic regression analysis. Getz and Weinstein^7^ used analyses of variance and multiple range tests and Kendall’s correlation tests. In Lenchner’s^5^ study only chi-squared test was used.

In order to assess intra-examiner agreement, Aminabadi et al^4^ used paired-samples t-test. Inter-observer calibration was calculated using kppa score in Davidovich et al^8^ study. Inter-judge agreement was established by Pearson's r in the study by Getz and Weinstein.^7^

## Discussion

The aim of this systematic review was to establish a comprehensive picture of the effect of treatment duration on child patients’ behavior and/or anxiety during or after dental treatment, and to clarify the inconsistency around the issue. A child’s behavior in dental situation is a result of interconnected relations between personal characteristics and situational and environmental factors.^10-12^ Treatment duration is a central situational factor which causes deterioration of children’s behavior during or after dental treatment.^10,13,14^ However, our review demonstrated that treatment duration has been largely overlooked in pediatric dental literature given that there were only 4 studies over the past 50 years that investigated the effect of treatment duration on children’s behavior.^4,5,7,8^ Therefore, there is a serious need to further investigate how treatment duration influences the child’s behavior or anxiety during and after treatment. Although the results are inconsistent to some extent, the overall direction of the findings of this review support the hypothesis that extended dental treatment length is associated with higher levels of behavioral problems in child patients.

There is no general agreement on the appropriate duration of treatment in child dental patients, and there is no study explicitly investigating the most appropriate length of dental treatment for children. Although Aminabadi et al^4^ suggested some treatment durations for children of different ages, it should be considered that these suggestions are based on arbitrary assumptions and should be confirmed in future studies. There is a need for studies in which the treatment duration is manipulated as the independent variable and children’s behavior and anxiety during and/or after treatment are assessed as the main outcome variables. In addition, the definition of long and short treatment/procedure duration should be clarified. Lenchner^5^ defined the short treatment duration as <30 minutes and long duration as >45 minutes. Davidovich et al^8^ considered treatment for >30 minutes as long appointment. However, Getz and Weinstein^7^ considered each treatment phase short when it was less than five minutes. This significant discrepancy may affect the interpretation of findings. In addition, the way by which the duration of treatment is related to children’s behavior in the reviewed studies should be considered. In this topic an interesting study is one performed by Lenchner,^5^ where children’s behavioral differences on the dental chair were assessed during long versus short appointments. Although the procedures were different for each child in their study, such designs in which the duration of treatment is manipulated to detect changes in children’s behavior may be the most appropriate.

### Treatment duration and behavior of children with different ages

Even without a complete agreement among studies, the main results found in this review showed that younger children are more likely to demonstrate negative behavior with an increase in treatment duration. Aminabadi et al^4^ provided a framework within which to consider the effect of treatment duration on children with different ages. Their findings showed a significant deterioration of behavior in younger children as treatment time was extended.

Age is a well-established factor which determines children’s behavior during treatment. In fact, the child’s stage of development determines their ability to cope with dental procedures and it seems that older children have greater ability to comply with the dental treatment.^13,15,16^ Most of the included studies have focused on the preschool age group, who often exhibit behavior problems and dental anxiety, requiring an increased time during dental treatment.^10^ Conversely, school-aged children may have developed sufficient coping skills to comply with the treatment when appropriate communication is established by the clinician.^7,10^ Therefore, in order to eliminate the effect of age as a confounding variable, studies should include and investigate children with similar age, or at least in the same developmental stage.

The attention span of children in various ages is also an important determinant of their reaction to treatment length.^4^ Children generally have difficulty sustaining attention for long periods of time. Attention problems are common among preschool children and progressively develop until the late school ages. By planning a variety of clinical activities based on the child’s age and attention span, the practitioner is more likely to maintain the child’s attention and limit inappropriate behavior.^17,18^ Furthermore, given these considerations, development of valid objective methods for assessing attention in preschool children could be particularly important.

### Treatment duration and children’s dental fear and anxiety

All the reviewed studies have focused on children’s behavior, ignoring fear and anxiety during treatment. Anxiety and fear can be major barriers for children to accept and tolerate treatment. They are relatively common occurrences in the dental setting and may lead to BMP during treatment.^9,19^ It has been reported that children with high dental fear also have other behavioral and emotional problems.^17,20^ On the other hand, children’s behavior reflects their inability to cope with anxiety and fear, and behavior management techniques provide children with appropriate coping strategies.^16^ It is assumed that anxiety impairs children’s attention system and increases the extent to which they react to threatening stimuli by reducing the ability to inhibit incorrect prepotent responses.^21,22^ Therefore, the importance of dental fear and anxiety in developing BMP and, consequently, interrupting treatment procedure should be considered as a central potential mediating factor during dental procedures for children.^19,23^

The distinction between dental fear, dental anxiety and BMP is also important.^19^ Children with dental fear/anxiety may express uncooperative behavior and vice versa.^24^ Therefore, in order to conduct a study related to the effect of treatment duration in children, the inclusion of children should be made by accurate identification of the existence of dental fear/anxiety or a history of BMP by incorporating appropriate measures into diagnostic protocols. Considering the fact that these conditions may affect children’s behavior during and after dental treatment, children with dental fear/anxiety or history of BMP should be excluded.^25^ In addition, the children’s mental health status should also be considered as performed by Aminabadi et al^4^ in their study. Since neuropsychiatric disorders affect at least 5% of the child population, it is likely that dentists frequently meet children and adolescents with these disorders, who may present with BMP or fear/anxiety as part of their diseases.^17^ Therefore, an adequate screening focused on the problems within the neuropsychiatric spectrum should be routinely performed in studies, especially those with referred patients.

### Treatment duration and children’s history of BMP

Davidovich et al^8^ evaluated children who had uncooperative behavior prior to treatment. Children’s dental fear/anxiety and history of BMP are confounding factors when treatment duration, as the cause of disruptive behaviors, is assessed among children. Moreover, it is possible that as the fear- or anxiety-related behavior increases, the length of the treatment increases presumably because of uncooperative behaviors by the child and vice versa. Interestingly, it has been noted that treatment duration of patients with anxiety or BMP is on average 40% more than that of normal patients going through the same procedure.^7^ Therefore, dentists should consider conditioning and gradual exposure to obtain cooperation in young children, and to maintain their cooperation by making the treatment as short as possible. In addition, dental practitioners could adopt a time-out policy. Although the degree of success of time-out varies, it appears to decrease patient disruptiveness in selected cases. In addition, time-out period and the frequency of time-out episodes are important considerations for its effectiveness. The time-out should be short and only be used once or twice to gain the acceptable behavior in any given child; otherwise, its failure is possible.^26^

### Treatment duration and operator’s clinical experience

The dentist’s clinical experience is also essential for establishing the adequate duration for each child’s treatment. The origin of BMP in some children is primarily due to their previous negative experiences with dental care.^27^ Well-trained and experienced pediatric dentists may be more efficient in providing treatment in shorter course and with the least traumatic approaches which significantly contribute to the children’s behavior during and after treatment.^3^ Of the reviewed studies, in Aminabadi et al study^4^ a pediatric dentist served as the operator and performed treatment procedures. In the study by Getz and Weinstein^7^, 22 general practitioners and three pedodontists participated in the study. Davidovich et al study^8^ was conducted in a postgraduate clinic and private practices of two of the authors.

### Complexity of treatment and interaction with the length of treatment and children’s behavior

The extent of delivered treatment and associated pain should be considered as they may interact with the length of treatment and children’s behavior during or after treatment.^28^ Davidovich et al^8^ classified complexity of treatments in their study as simple procedures, including fissure sealants, prophylaxis, Class I and Class II restorations, and as complex, including stainless steel crowns, pulp therapy and dental extractions. Relying on the above-mentioned classification, in Aminabadi et al^4^ and Getz and Weinstein study^7^ the participants received restorative treatments which can be considered as simple procedures. Lenchner^5^ provided both simple and complex treatments for all the participants in two consecutive sessions, which allowed better understanding of the interplay between treatment type and children’s behavior during treatment. However, the complexity of treatment usually has no correlation with the duration of treatment. For instance, dental extraction is considered a complex treatment, although it was obviously shorter than a Class I restoration. Therefore, it is better to consider invasiveness of treatment in future studies as it may better present the nature of treatment.

### Methodological issues

This review confirmed the effect of the procedure length on children’s behavior during dental treatment. However, all the 4 studies have their weaknesses in methodology, design and data analysis. Most of the studies had a relatively small sample size. Except for the study by Aminabadi et al,^4^ which included 450 children with a wide age range, the remaining studies had a sample size of <100 participants. Apparently, there is a lack of representative and population-based samples to understand potential differences between various treatments, ages and genders. In addition, none of the studies included sample size calculation, and participant recruitment procedures varied considerably among the studies. Future studies in this field should consider representative sampling, recruitment standardization and justification of sample size to test the associations with sufficient power.

Besides, concerns were raised on the BMP assessment quality due to poor assessors' (parents or dentists) knowledge level on this psychological matter, and the lack of inter-rater reliability. In Lenchner’s study,^5^ parents and dentists were asked to rate the children’s behavior using one simple question. The frequencies of observed behaviors by dentists were reported in Getz et al study^7^ as a dichotomous variable, which may lead to elimination or masking of certain important details. Aminabadi et al^4^ used two observers to evaluate children’s behavior during treatment, using sound, eye and motor (SEM) scale with high inter-rater reliability. In Davidovich et al study,^8^ children’s behavior during and following treatment were obtained from the records of children. As reported above, there is a relevant incoherence among the included studies in both the methods and measurement tools to rate the children’s behavior. In addition, it has been noted that the assessment of behaviors over a short period of time is more accurate than long period of time.^29^ Therefore, in order to reach a high correlation between ratings and behavior, the dentists should rate behavior within a relatively short period of time (6‒30 minutes).^30^

All the included studies adopted traditional analysis methods of the data obtained from observers’ ratings or grading on scales. The use of lag sequential analysis technique is advocated to identify patterns of behavior of children. In addition, the adoption of autocorrelation in the lag sequential analysis may further advance our understanding as the occurrence of certain behavior is not influenced only by treatment duration.^31^ Moreover, no study considered the clustering effect of personal characteristics and situational and environmental factors. In order to take account of some participant-specific variables, attempts to control for clustering (such as multilevel modeling) should be incorporated into sequential analysis. The traditional multivariate variable-based approach to investigate the relationships among variables does not fully explain the interplay between variables within each child. To understand the cumulative effect of all variables on each child, analyses with a person-based approach are valuable complements. In such analyses, children are studied on the basis of their unique pattern (profile) of values for variables that are relevant to the research question.^32^

### Implications for current practice and conclusion

Our review confirmed the existence of a relationship between treatment duration and children’s behavior in an age-dependent manner. However, the various problems surrounding the issue make the identification of optimal appointment length in pediatric dentistry problematic and undermine the validity of current suggestions about appropriate treatment duration. Our review demonstrated that there are a number of significant gaps in the current literature, including the differences among studies related to culture, study design and methodology, sampling methods and sizes, as well as the inhomogeneous measurement procedures of both dental anxiety and BMP affecting the relevance of the findings. Therefore, comprehensive clinical investigations on how the treatment length influences child’s anxiety and behavior during and after treatment are recommended. The focus not only on single behavioral elements but also on multidimensional assessment of children, including emotional, behavioral, cognitive and physiological components and response systems, may prove to be beneficial. In addition further research is needed to develop consistency in the assessment of child anxiety and behavior with sophisticated and valid behavioral codes.

## Acknowledgments

The authors would like to thank the staff in the library of Faculty of Dentistry for providing full-texts of papers.

## Authors’ contributions

NAA and SS contributed to the conception and design, and critically revised the manuscript. ASD, ZJ, EN, ZEA and SS contributed to data acquisition and interpretation, and drafted the manuscript. SS consulted on and performed statistical evaluation. EN and SS contributed substantially to discussion. All the authors gave final approval and agree to be accountable for all the aspects of the work ensuring integrity and accuracy.

## Funding

This study was supported and funded by Dental and Periodontal Research Center, Faculty of Dentistry, Tabriz University of Medical Sciences.

## Competing interests

The authors declare no competing interests with regards to the authorship and/or publication of this article

## References

[1] Hotz S, Kaptein A, Pruitt S, Sánchez-Sosa J, Wiley C, WHO. Behavioural mechanisms explaining adherence: What every health professional should know. Adherence to long term therapies: Evidence for action. 2003:135-49.

[2] Erfanparast L, Vafaei A, Sohrabi A, Ranjkesh B, Bahadori Z, Pourkazemi M (2015). Impact of Self-concept on Preschoolers' Dental Anxiety and Behavior. J Dent Res Dent Clin Dent Prospects.

[3] Aminabadi NA, Najafpour E, Aghaee S, Sighari‏ Deljavan A, Jamali Z, Shirazi S (2016). Use of general anaesthesia in paediatric dentistry: barriers to discriminate between true and false cases. Eur Arch Paediatr Dent.

[4] Aminabadi NA, Oskouei SG, Farahani RM‏ (2009). Dental treatment duration as an indicator of the behavior of 3-to 9-year-old pediatric patients in clinical dental settings. J Contemp Dent Pract.

[5] Lenchner V (1966). The effect of appointment length on behavior of the pedodontic patient and his attitude toward dentistry. J Dent Child.

[6] Wright GZ, Alpern GD (1971). Variables influencing children's cooperative behavior at the first dental visit. ASDC J Dent Child.

[7] Getz T, Weinstein P (1981). The effect of structural variables on child behavior in the operatory. Pediatr Dent.

[8] Davidovich E, Wated A, Shapira J, Ram D (2013). The influence of location of local anesthesia and complexity/duration of restorative treatment on children's‏ behavior during dental treatment. Pediatr Dent.

[9] Klingberg G, Broberg AG (1998). Temperament and child dental fear. Pediatr Dent.

[10] Pai R, Mandroli P, Benni D, Pujar P (2015). Prospective analysis of factors associated with dental behavior management problems, in children aged 7-11 years. J Indian Soc Pedod Prev Dent.

[11] Najafpour E, Asl-Aminabadi N, Nuroloyuni S, Jamali Z, Shirazi S (2017). Can galvanic skin conductance be‏ used as an objective indicator of children's anxiety in the dental setting?. J Clin Exp Dent.

[12] Jamali Z, Vatandoost M, Erfanparast L, Aminabadi NA, Shirazi S (2018). The relationship between children's media habits and their anxiety and behaviour during dental treatment. Acta Odontol Scand.

[13] Suprabha BS, Rao A, Choudhary S, Shenoy R (2011). Child dental fear and behavior: the role of environmental factors in a hospital cohort‏. J Indian Soc Pedod Prev Dent.

[14] Gustafsson A, Arnrup K, Broberg AG, Bodin L, Berggren U (2007). Psychosocial concomitants to dental fear and behaviour management problems. Int J Paediatr Dent.

[15] Mirzakouchaki B, Shirazi S, Sharghi R, Shirazi S (2016). Assessment of Factors Affecting Adolescent Patients' Compliance with Hawley and Vacuum Formed Retainers. J Clin Diagn Res.

[16] Gupta A, Marya CM, Bhatia HP, Dahiya V (2014). Behaviour management of an anxious child. Stomatologija.

[17] Aminabadi NA, Najafpour E, Erfanparast L, Jamali Z, Pournaghi-Azar F, Tamjid-Shabestari S (2016). Oral health status, dental anxiety, and behavior-management problems in children with oppositional defiant disorder. Eur J Oral Sci.

[18] Palfrey JS, Levine MD, Walker DK, Sullivan M (1985). The emergence of attention deficits in early childhood: a prospective study. J Dev Behav Pediatr.

[19] Aminabadi NA, Shokravi M, Jamali Z, Shirazi S (2017). Barriers and Drawbacks of the Assessment of Dental Fear, Dental Anxiety and Dental Phobia in Children: A Critical Literature Review. J Clin Pediatr Dent.

[20] ten Berge M, Veerkamp JS, Hoogstraten J, Prins PJ (1999). Behavioural and emotional problems in children referred to a centre for special dental care. Community Dent Oral Epidemiol.

[21] Eysenck MW, Derakshan N, Santos R, Calvo MG (2007). Anxiety and cognitive performance: attentional control theory. Emotion.

[22] Zeidner M, Matthews G. Anxiety 101: Springer Publishing‏ Company; 2010‏.

[23] Aminabadi NA, Ghoreishizadeh A, Ghoreishizadeh M, Oskouei SG (2011). Can drawing be considered a projective measure for children's distress in paediatric dentistry?. Int J Paediatr Dent.

[24] Klinberg G (2008). Dental anxiety and behaviour management problems in paediatric dentistry--a review of background factors and diagnostics. Eur Arch Paediatr Dent.

[25] Klingberg G, Broberg AG (2007). Dental fear/anxiety and dental behaviour management problems in children and adolescents: a review of prevalence and concomitant psychological factors. Int J Paediatr Dent.

[26] McTigue DJ, Fields HW, Pinkham JR, Casamassimo PS. Pediatric Dentistry: Infancy through Adolescence, 5/e: Elsevier India; 2013‏.

[27] Carrillo-Diaz M, Crego A, Armfield JM, Romero-Maroto M (2012). Treatment experience, frequency of dental visits, and children's dental fear: a cognitive approach. Eur J Oral Sci.

[28] de Menezes Abreu DM, Leal SC, Mulder J, Frencken JE (2011). Pain experience after conventional, atraumatic, and ultraconservative restorative treatments in 6- to 7-yr-old children. Eur J Oral Sci.

[29] Allen KD, Stanley RT, McPherson K (1990). Evaluation of behavior management technology dissemination in pediatric dentistry. Pediatr Dent.

[30] Allen KD, Hutfless S, Larzelere R (2003). Evaluation of‏ two predictors of child disruptive behavior during restorative dental treatment. J Dent Child (Chic).

[31] Zhou Y, Cameron E, Forbes G, Humphris G (2011). Systematic review of the effect of dental staff behaviour on child dental patient anxiety and behaviour. Patient Educ Couns.

[32] Arnrup K, Broberg AG, Berggren U, Bodin L (2002). Lack of cooperation in pediatric dentistry--the role of child personality characteristics. Pediatr Dent.

